# Peripheral Blood Neutrophil–Monocyte-to-Lymphocyte Ratio and Long-Term All-Cause Mortality in Patients with Chronic Obstructive Pulmonary Disease: A Population-Based Primary Care Cohort Study

**DOI:** 10.3390/healthcare14152431

**Published:** 2026-08-06

**Authors:** Josep Montserrat-Capdevila, Pilar Vaqué Castilla, Albert Romero Gracia, Jennyfer Jiménez-Díaz, Joan Deniel-Rosanas, Araceli Fuentes, Eugeni Paredes, Mònica Solanes-Cabús, Sofia Godoy, Sandra Moreno Garcia, Joaquim Sol, Meritxell Calderó, Cristina Farràs, Montserrat Gea-Sánchez, Laia Llubes-Arrià, Jacob Mesalles, Maria Teresa Castañ-Abad, José María Palacín Peruga, Josep Vidal-Alaball, Antoni Sisó-Almirall, Pere Godoy

**Affiliations:** 1 Teaching Unit of Family and Community Care, Primary Care and Community Health, Catalan Health Institute (ICS), 25007 Lleida, Spain; 2 Primary Care Center Mollerussa, Catalan Health Institute (ICS), 25230 Mollerussa, Spain; 3 Catalan Society of Family and Community Medicine (CAMFiC), 08009 Barcelona, Spain; 4 Biomedical Research Institute of Lleida (IRBLleida), University of Lleida, 25198 Lleida, Spain; 5 University of Lleida, 25008 Lleida, Spain; 6 Fundació Institut Universitari per a la Recerca a l’Atenció Primària de Salut Jordi Gol i Gurina (IDIAPJGol), 08007 Barcelona, Spain; 7 Primary and Community Health Care Management (GAPiC), Catalan Health Institute (ICS), 25007 Lleida, Spain; 8 Primary Care Center Onze de Setembre, Catalan Health Institute (ICS), 25005 Lleida, Spain; 9 Clinical Assessment Unit, Primary and Community Health Care Management (GAPiC), Catalan Health Institute (ICS), 25007 Lleida, Spain; 10 Primary Care Center Eixample, Catalan Health Institute (ICS), 25006 Lleida, Spain; 11 Primary Care Center Balàfia, Catalan Health Institute (ICS), 25005 Lleida, Spain; 12 Primary Care Center Rambla Ferran, Catalan Health Institute (ICS), 25007 Lleida, Spain; 13 Faculty of Medicine, University of Vic–Central University of Catalonia, 08500 Vic, Spain; 14 Unitat de Recerca i Innovació, Gerència d’Atenció Primària i a la Comunitat de la Catalunya Central, Institut Català de la Salut, 08242 Manresa, Spain; 15 Intelligence for Primary Care Research Group, Fundació Institut Universitari per a la Recerca a l’Atenció Primària de Salut Jordi Gol i Gurina, 08242 Manresa, Spain; 16 Consortium for Biomedical Research in Epidemiology and Public Health (CIBERESP), 28029 Madrid, Spain; 17 Population Cancer Registry, University Hospital of Santa Maria, 25198 Lleida, Spain

**Keywords:** chronic obstructive pulmonary disease, mortality, inflammatory biomarkers, neutrophil-to-monocyte plus lymphocyte ratio, risk stratification, primary care, precision medicine, prognosis

## Abstract

**Highlights:**

**What are the main findings?**

- Among 2644 patients with spirometry-confirmed COPD and available baseline neutrophil-to-monocyte plus lymphocyte ratio (NMLR), 1085 deaths occurred during 19,231 person-years of follow-up.
- Elevated baseline NMLR was independently associated with increased long-term all-cause mortality, with patients in the highest quartile showing an approximately twofold-higher adjusted risk compared with those in the lowest quartile.

**What are the implications of the main findings?**

- Higher baseline NMLR was independently associated with increased long-term all-cause mortality in patients with COPD.
- Baseline NMLR may improve mortality risk stratification and contribute to precision follow-up approaches in primary care, although external validation is required before clinical implementation.

**Abstract:**

**Background/Objectives:** Chronic obstructive pulmonary disease (COPD) is a heterogeneous syndrome with substantial variability in clinical progression and survival. Although neutrophil-to-lymphocyte ratio has been widely studied, evidence regarding neutrophil-to-monocyte plus lymphocyte ratio (NMLR) remains limited. This study evaluated whether baseline peripheral blood neutrophil-to-monocyte plus lymphocyte ratio (NMLR), a composite marker of systemic inflammation, was independently associated with long-term all-cause mortality in patients with COPD managed in primary care. **Methods:** We conducted a retrospective population-based cohort study using electronic health records from the Lleida Health Region (Spain) between 1 January 2014 and 31 December 2023. Patients with spirometry-confirmed COPD and available baseline complete blood counts were included. Baseline NMLR was calculated as (absolute neutrophil count + absolute monocyte count)/absolute lymphocyte count using the first valid blood sample obtained after cohort entry. Patients were categorized into NMLR quartiles. Associations with all-cause mortality were assessed using multivariable Cox proportional hazards regression models adjusted for age, sex, smoking status, lung function, and baseline comorbidity burden. **Results:** A total of 2644 patients were included, contributing 19,231 person-years of follow-up. During a median follow-up of 8.8 years, 1085 deaths occurred. Mortality increased progressively across NMLR quartiles, from 28.3% in the lowest quartile to 60.2% in the highest quartile. In fully adjusted analyses, patients in the highest quartile had higher all-cause mortality than those in the lowest quartile (hazard ratio [HR] 2.00; 95% confidence interval [CI] 1.66–2.40; *p* < 0.001). When modeled continuously, each doubling of baseline NMLR was associated with a 34% increase in mortality risk (HR 1.34; 95% CI 1.26–1.42). **Conclusions:** Elevated baseline NMLR was independently associated with increased long-term mortality in COPD. As an inexpensive and routinely available inflammatory biomarker, NMLR may improve mortality risk stratification and support future evaluation of risk-stratified follow-up strategies in primary care. External validation is required before implementation in routine clinical practice.

## 1. Introduction

Chronic obstructive pulmonary disease (COPD) remains one of the leading causes of morbidity and mortality worldwide and represents a major public health challenge because of its high prevalence, progressive functional impairment, and heterogeneous clinical trajectories [1,2,3]. Recent Global Initiative for Chronic Obstructive Lung Disease (GOLD) reports recognize COPD as a complex and heterogeneous respiratory condition extending beyond airflow limitation alone [1]. Despite advances in pharmacological and non-pharmacological management, considerable interindividual variability persists in disease progression, exacerbation burden, treatment response, and survival, highlighting the need for scalable prognostic biomarkers capable of supporting individualized follow-up.

Historically, COPD severity and prognosis have been largely defined using spirometric measures, particularly forced expiratory volume in one second (FEV1). However, airflow limitation alone only partially captures the biological and clinical complexity of COPD and is insufficient to explain long-term outcomes [1,4]. COPD is increasingly recognized as a multidimensional syndrome shaped by pulmonary injury, ageing, environmental exposures, host susceptibility, extrapulmonary manifestations, and comorbidity burden [2,3,4]. This concept has promoted precision medicine and the treatable-traits approach, which seek to identify measurable clinical and biological characteristics that improve risk stratification beyond spirometry [5,6,7].

Among the biological mechanisms contributing to COPD heterogeneity, systemic inflammation has emerged as a major determinant of adverse outcomes [8,9,10]. Persistent inflammatory activation has been associated with exacerbations, comorbidity burden, functional decline, and mortality [8,9,10]. Consequently, circulating inflammatory biomarkers have attracted increasing interest in risk stratification and phenotype characterization.

Several blood-based biomarkers, including C-reactive protein, fibrinogen, blood eosinophils, neutrophil-to-lymphocyte ratio (NLR), monocyte-to-lymphocyte ratio (MLR), platelet-to-lymphocyte ratio (PLR), systemic immune-inflammation index (SII), systemic inflammation response index (SIRI), and other monocyte-derived indices, have been evaluated in COPD [9,10,11,12]. Although some have shown associations with exacerbation risk, treatment response, and mortality, their incorporation into routine clinical practice remains limited because of heterogeneous study designs, variable thresholds, limited incremental predictive value, and insufficient validation in real-world populations [10,11,12]. Therefore, inexpensive biomarkers derived from routinely available laboratory tests remain of particular interest.

The neutrophil-to-monocyte plus lymphocyte ratio (NMLR), calculated as the sum of absolute neutrophil and monocyte counts divided by the absolute lymphocyte count, combines markers of innate immune activation with adaptive immune regulation [13]. Neutrophils contribute to airway inflammation and monocytes to chronic inflammatory amplification, whereas lymphocyte depletion may reflect impaired immune regulation. Consequently, elevated NMLR may identify a systemic inflammatory phenotype with prognostic relevance. By integrating neutrophil, monocyte, and lymphocyte counts into a single composite index, NMLR may provide a broader representation of systemic inflammatory and immune dysregulation than indices based on only two leukocyte subpopulations. However, whether this biological rationale translates into incremental prognostic value over other inflammatory indices remains uncertain and requires direct comparative studies.

Although neutrophil-to-lymphocyte ratio (NLR) has been extensively investigated as an inflammatory prognostic biomarker in COPD, evidence regarding neutrophil-to-monocyte plus lymphocyte ratio (NMLR) remains limited. Recent evidence suggests that NMLR may predict adverse outcomes in chronic inflammatory diseases, including COPD [13]. In a retrospective COPD cohort, higher peripheral blood NMLR was associated with increased five-year all-cause mortality [13]. However, current evidence is limited and derives mainly from selected hospital-based populations, frequently including patients with acute exacerbations. Data evaluating baseline NMLR in stable, population-based primary care cohorts remain scarce.

Primary care is an especially relevant setting for evaluating prognostic biomarkers because it includes a broad spectrum of COPD severity, multimorbidity, and frailty, where most long-term follow-up takes place. In addition, complete blood counts are inexpensive, routinely available, and easily applicable in clinical practice, making NMLR an attractive candidate biomarker if its prognostic value is confirmed.

Therefore, the present study aimed to evaluate whether baseline peripheral blood NMLR is independently associated with long-term all-cause mortality in a population-based cohort of patients with spirometry-confirmed COPD followed in primary care over a ten-year period. We hypothesized that elevated baseline NMLR identifies a systemic inflammatory phenotype associated with increased mortality independently of demographic characteristics, smoking exposure, lung function, and comorbidity burden.

## 2. Materials and Methods

### 2.1. Study Design and Data Source

This retrospective population-based cohort study was conducted using routinely collected electronic health record (EHR) data from primary care centers in the Lleida Health Region (Catalonia, Spain). The study period extended from 1 January 2014 to 31 December 2023. The EHR system includes longitudinal information recorded during routine clinical care, including demographic characteristics, diagnoses, spirometry results, laboratory measurements, and mortality data.

### 2.2. Study Population

The source population consisted of 16,898 individuals with a recorded diagnosis of chronic obstructive pulmonary disease (COPD) identified in the extracted dataset. For the present study, analyses were restricted to patients fulfilling spirometric criteria for COPD according to the predefined extraction procedure. Eligible spirometry was defined as a recorded FEV1/FVC ratio < 0.70 obtained between 1 January 2012 and 31 December 2013. Patients were additionally required to have at least one valid differential blood count available after 1 January 2014 to allow for calculation of the exposure variable. Patients were excluded if leukocyte measurements required for exposure assessment were unavailable or invalid or if the recorded date of death occurred before exposure assessment. The final analytic cohort included 2644 patients.

### 2.3. Exposure Variable

The primary exposure was baseline neutrophil-to-monocyte plus lymphocyte ratio (NMLR), calculated as NMLR = (absolute neutrophil count + absolute monocyte count)/absolute lymphocyte count. Absolute cell counts were obtained from routine peripheral blood analyses recorded in the EHR database. For each participant, the most recent valid complete blood count available around cohort entry and obtained under clinically stable conditions was selected as the baseline measurement. Blood counts performed during hospitalization, acute infection, COPD exacerbation, or decompensation of another chronic condition were not considered representative of baseline status. NMLR was evaluated both as a categorical variable using cohort-specific quartiles and as a continuous variable after log2 transformation to estimate the relative effect associated with each doubling of baseline NMLR values.

### 2.4. Outcome

The primary and only outcome of interest was all-cause mortality. Follow-up started at the date of baseline NMLR assessment and continued until death or administrative censoring on 31 December 2023, whichever occurred first. Follow-up time was calculated individually and expressed as person-years.

The available database provided mortality status and date of death but did not contain a complete and validated underlying cause of death for all participants. Therefore, the outcome was defined as all-cause mortality, and cause-specific mortality analyses were not performed.

### 2.5. Covariates

Covariates were defined before statistical modelling according to clinical relevance and availability in the extracted dataset. The multivariable analysis included age, sex, smoking status, and lung function expressed as forced expiratory volume in one second percentage predicted (FEV1% predicted). Baseline comorbidities recorded before exposure assessment were additionally considered, including hypertension, diabetes mellitus, ischemic heart disease, heart failure, atrial fibrillation, chronic kidney disease, cancer, and bronchiectasis. Body mass index (BMI) and Charlson Comorbidity Index contained a substantial proportion of missing values and were therefore not included in the primary complete-case multivariable model. As a sensitivity analysis, missing values were handled using Multiple Imputation by Chained Equations (MICE), generating 20 imputed datasets. The multivariable Cox regression model was then repeated after additional adjustment for BMI and Charlson Comorbidity Index.

### 2.6. Statistical Analysis

Continuous variables are reported as mean and standard deviation (SD) or median and interquartile range (IQR), as appropriate. Categorical variables are presented as absolute frequencies and percentages. Continuous variables were compared using one-way analysis of variance (ANOVA) or Kruskal–Wallis tests, as appropriate. Categorical variables were compared using χ^2^ tests. Mortality incidence rates were calculated as deaths per 100 person-years of follow-up. Estimated survival probabilities were calculated using the Kaplan–Meier method according to baseline NMLR quartiles and compared using the log-rank test. Associations between baseline NMLR and all-cause mortality were evaluated using Cox proportional hazards regression models and expressed as hazard ratios (HRs) with 95% confidence intervals (CIs). The proportional hazards assumption was formally assessed using Schoenfeld residuals and graphical diagnostics before fitting the final Cox regression models. No evidence of meaningful violation of the proportional hazards assumption was identified. Three models were estimated: an unadjusted model, a model adjusted for age and sex, and a multivariable model additionally adjusted for smoking status, FEV1% predicted, and baseline comorbidities. For continuous analyses, NMLR values were log2-transformed and interpreted as the relative change in mortality associated with each doubling of NMLR. All statistical analyses were performed using Python version 3.13. Data management and preprocessing were conducted using pandas (version 2.2.3) and NumPy (version 2.2.6). Descriptive statistical analyses and hypothesis testing were performed using SciPy (version 1.15.3). Survival analyses and Cox proportional hazards regression models were performed using lifelines (version 0.30.0). Multiple imputation by chained equations (MICE) was performed using scikit-learn (version 1.7.0). All statistical tests were two-sided, and statistical significance was defined as *p* < 0.05.

Sensitivity analysis. To assess the potential impact of missing data, a sensitivity analysis was performed using Multiple Imputation by Chained Equations (MICE). Twenty imputed datasets were generated for missing BMI and Charlson Comorbidity Index values. The imputation model included all variables entered into the primary multivariable Cox regression model, together with follow-up time and mortality status. The multivariable Cox regression model was subsequently repeated with additional adjustment for BMI and Charlson Comorbidity Index. Estimates from the imputed datasets were pooled according to Rubin’s rules.

### 2.7. Ethical Considerations

This study was conducted in accordance with the ethical principles of the Declaration of Helsinki and applicable regulations governing observational research using routinely collected healthcare data. The study protocol was reviewed and approved by the Research Ethics Committee with Medicines (Comitè d’Ètica d’Investigació amb Medicaments, CEIm) of the IDIAP J Gol, Barcelona, Spain (approval code: 23/280-P; approved on 28 May 2025). The study was based on secondary analysis of routinely collected electronic health record data. Data extraction and analysis were performed using anonymized or pseudonymized information in accordance with institutional procedures and applicable data protection regulations. Given the retrospective observational design and the use of routinely collected clinical data, informed consent was waived by the Ethics Committee.

## 3. Results

### 3.1. Cohort Derivation

**Figure 1:**
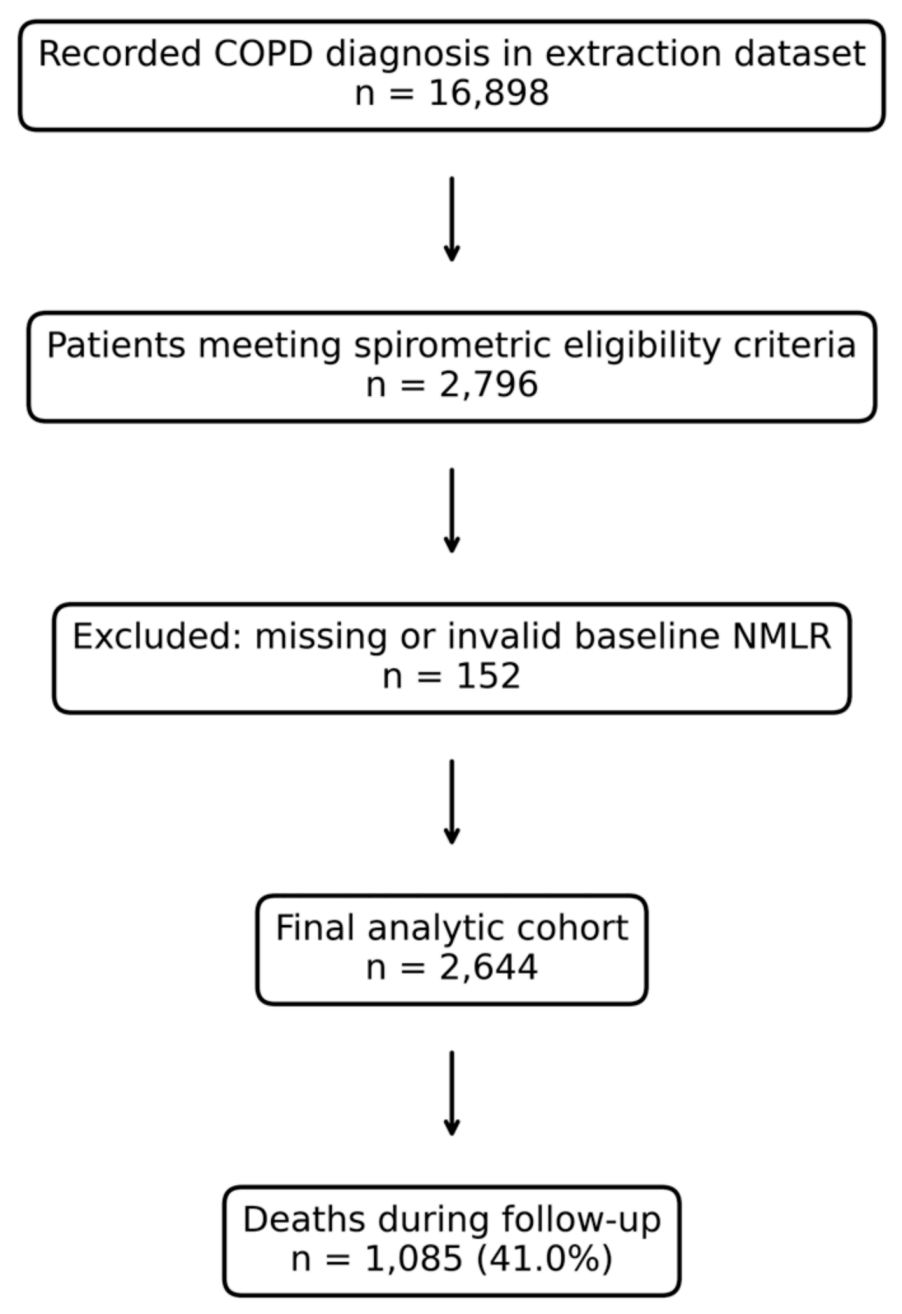
Flow diagram of cohort selection and derivation of the analytic cohort.

The extracted dataset included 16,898 individuals with a recorded diagnosis of chronic obstructive pulmonary disease (COPD). After restricting the population to patients fulfilling the predefined spirometric eligibility criteria (FEV1/FVC < 0.70 recorded between 1 January 2012 and 31 December 2013), 2796 patients remained eligible for analysis. Patients without a valid baseline neutrophil-to-monocyte plus lymphocyte ratio (NMLR) measurement were excluded from the analytic cohort. The final analytic cohort comprised 2644 patients with COPD confirmed by spirometry and available baseline NMLR values. During follow-up, 1085 deaths occurred over 19,231 person-years of observation, corresponding to an overall mortality proportion of 41.0%. Figure 1 summarizes the derivation of the analytic cohort. Of 2796 eligible patients, 152 were excluded because baseline NMLR values were unavailable.

### 3.2. Baseline Characteristics

Baseline characteristics stratified according to baseline NMLR quartiles are presented in Table 1. The mean age of the study population was 70.1 years (SD 11.5), and 25.2% of participants were women. Median baseline NMLR was 2.20 (IQR 1.63–3.09). Across increasing NMLR quartiles, patients tended to be older, more frequently male, and exhibited lower mean FEV1 percentage predicted values. Higher NMLR categories were also associated with a greater prevalence of selected comorbid conditions, particularly diabetes mellitus, heart failure, atrial fibrillation, chronic kidney disease, and cancer. Smoking status distribution showed limited variation across quartiles.

### 3.3. Mortality According to Baseline NMLR Quartile

**Figure 2:**
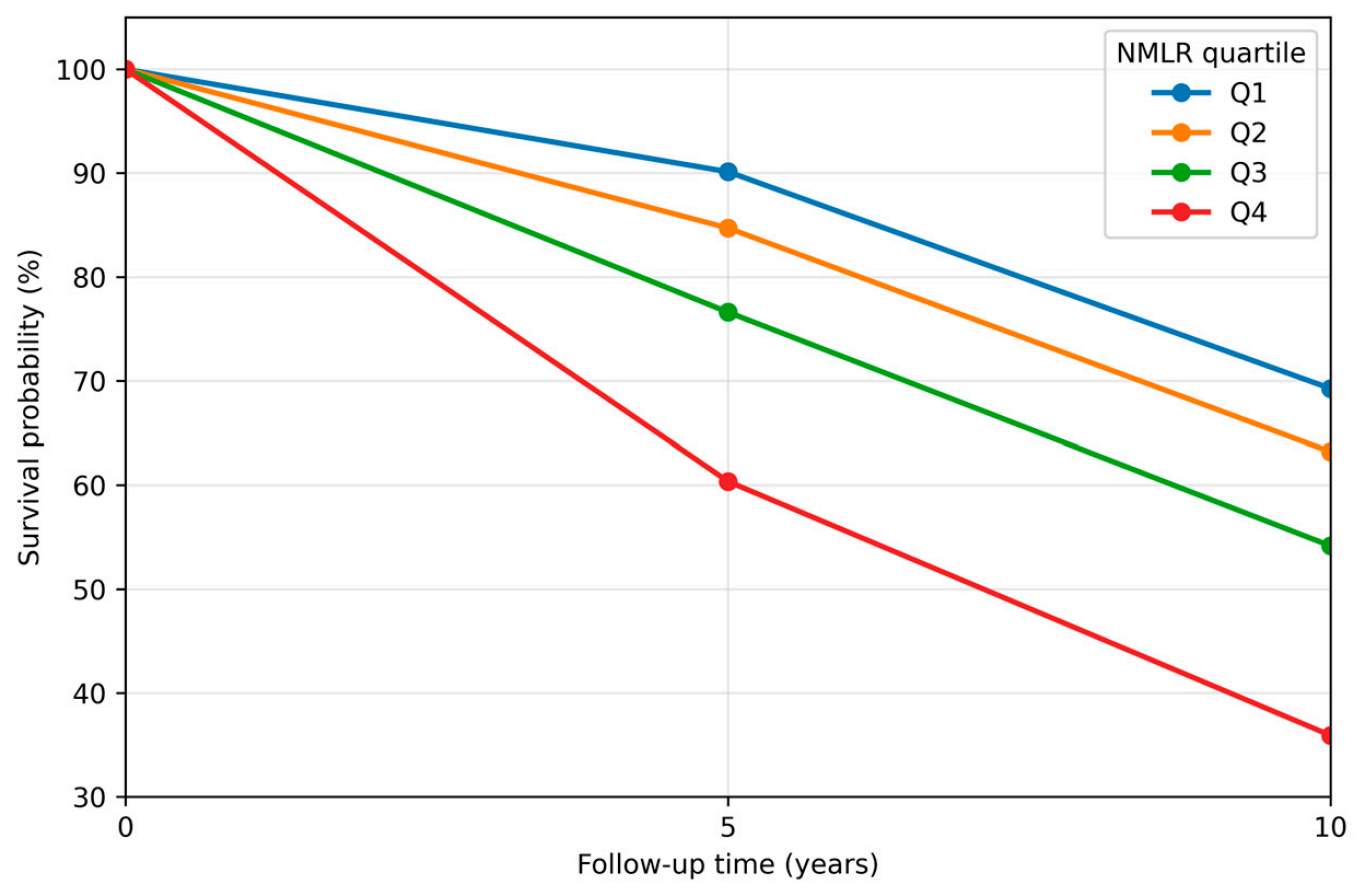
Kaplan–Meier survival curves according to baseline NMLR quartiles.

All-cause mortality increased progressively across baseline NMLR quartiles. Patients in the highest quartile exhibited the highest cumulative mortality, shortest median follow-up duration, and lowest estimated survival probabilities at both 5 and 10 years. Estimated survival probabilities demonstrated progressively lower survival across increasing baseline NMLR categories (Figure 2 and Table 2).

Estimated survival probabilities according to baseline NMLR quartiles are presented to visualize differences in survival over follow-up. Estimated survival probabilities decreased progressively across increasing baseline NMLR quartiles. Abbreviation: NMLR, neutrophil-to-monocyte plus lymphocyte ratio.

### 3.4. Association Between Baseline NMLR and All-Cause Mortality

Cox proportional hazards regression analyses showed progressively higher hazard ratios for all-cause mortality across increasing baseline NMLR quartiles. In unadjusted analyses, mortality risk increased progressively across quartiles. After adjustment for age and sex, the associations were attenuated but remained statistically significant for higher NMLR categories. In the fully adjusted model, the highest baseline NMLR quartile was associated with a twofold-higher hazard of all-cause mortality compared with the lowest quartile (HR 2.00; 95% CI 1.66–2.40; *p* < 0.001). When modeled continuously, each doubling of baseline NMLR was associated with a 34% increase in mortality hazard (HR 1.34; 95% CI 1.26–1.42; *p* < 0.001) (Table 3).

Model 3 was adjusted for age, sex, smoking status, FEV1% predicted, and baseline comorbidities. Continuous NMLR was analyzed after log_2_ transformation and interpreted as the relative change in mortality hazard associated with each doubling of baseline NMLR. The proportional hazards assumption was satisfied for all fitted Cox regression models. Abbreviations: CI, confidence interval; FEV1, forced expiratory volume in one second; HR, hazard ratio; NMLR, neutrophil-to-monocyte plus lymphocyte ratio.

#### Sensitivity Analysis

**Figure 3:**
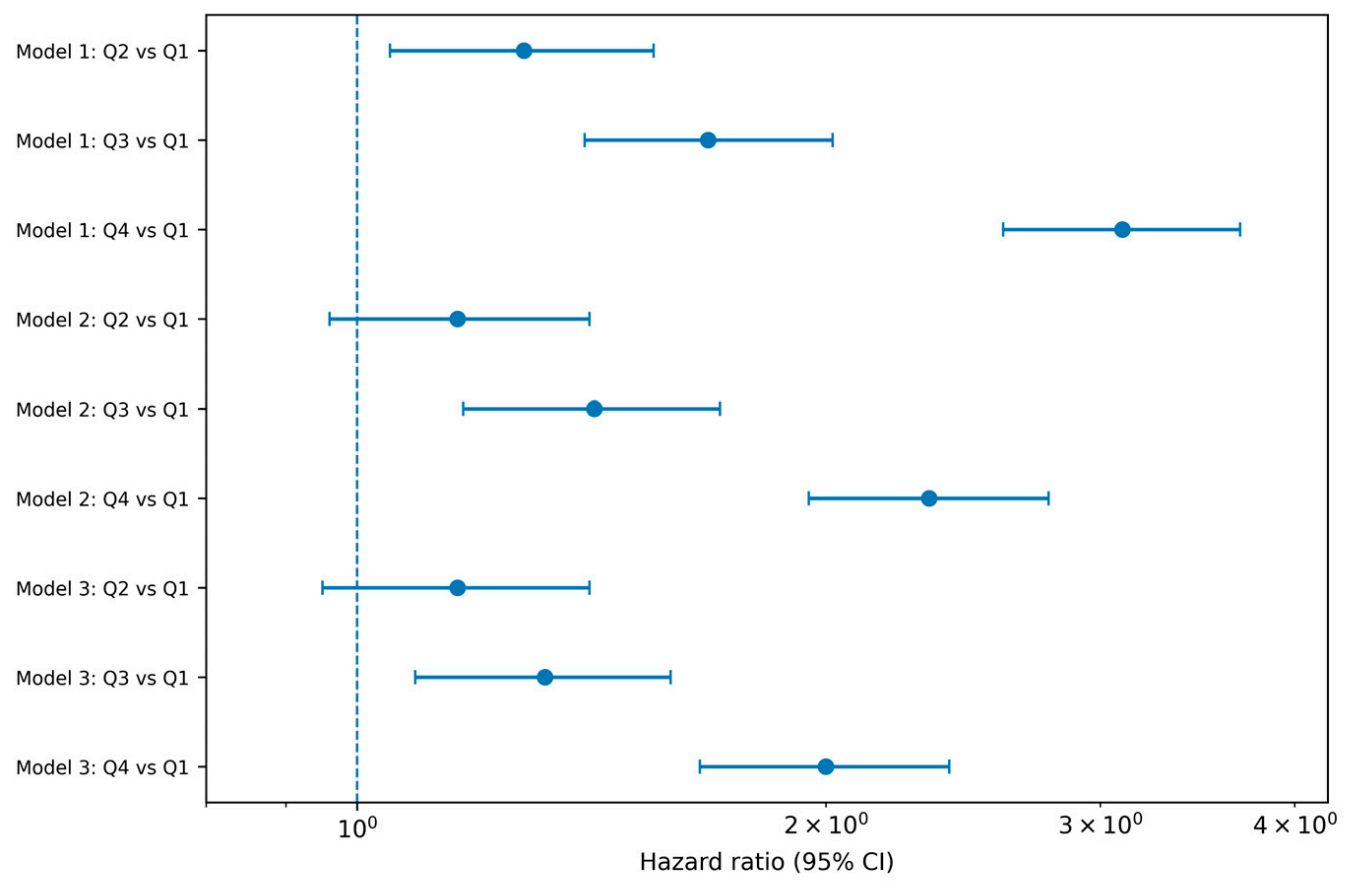
Hazard ratios for all-cause mortality according to baseline NMLR categories across Cox regression models.

A sensitivity analysis using Multiple Imputation by Chained Equations (MICE) was performed to account for missing BMI and Charlson Comorbidity Index values. After additional adjustment for these variables, the association between baseline NMLR and long-term all-cause mortality remained essentially unchanged compared with the primary complete-case analysis (Figure 3). The corresponding results are presented in Supplementary Table S1.

## 4. Discussion

In this population-based primary care cohort of patients with COPD confirmed by spirometry, higher baseline neutrophil-to-monocyte plus lymphocyte ratio (NMLR) values were independently associated with increased long-term all-cause mortality. Mortality increased progressively across NMLR quartiles, and patients in the highest quartile exhibited a twofold-higher adjusted hazard of death compared with those in the lowest quartile. Additionally, each doubling of baseline NMLR was associated with a 34% increase in mortality hazard after multivariable adjustment.

These findings support the initial study hypothesis and reinforce current concepts recognizing that COPD prognosis cannot be fully explained by airflow limitation alone [1,4,5]. Contemporary models increasingly describe COPD as a heterogeneous syndrome in which systemic biological processes contribute to differences in disease progression and survival [2,4,9,14].

Systemic inflammation has emerged as one of the mechanisms most consistently associated with adverse outcomes in COPD [9,10,11,15]. Previous longitudinal studies have shown associations between inflammatory activation and increased exacerbation burden, physical decline, cardiovascular comorbidity, and mortality [15,16,17]. Although the present study did not evaluate mechanistic pathways, the observed association between higher NMLR and mortality is compatible with prior evidence supporting a relationship between leukocyte-derived inflammatory markers and COPD prognosis.

Blood-derived inflammatory indices have gained interest because of their accessibility and low implementation cost [10,11,12,13,18,19]. Neutrophil-to-lymphocyte ratio (NLR) has been the most extensively studied index and has shown associations with mortality, exacerbations, and disease severity across several COPD populations [19,20,21]. More recently, interest has expanded toward composite leukocyte-derived indices integrating different components of innate and adaptive immunity [22,23]. In this context, NMLR may represent an extension of this approach, although evidence remains considerably more limited than for NLR.

From a biological perspective, the observed association is consistent with the current understanding of COPD as a disease characterized by persistent systemic inflammation and immune dysregulation. Neutrophils contribute to chronic inflammation through the release of proteases, reactive oxygen species, inflammatory cytokines, and neutrophil extracellular traps, whereas monocytes participate in chronic inflammatory signalling, macrophage differentiation, tissue remodelling, and the development of systemic comorbidities. Conversely, lower lymphocyte counts may reflect impaired adaptive immune responses and immunosenescence. By integrating neutrophil, monocyte, and lymphocyte counts into a single biomarker, NMLR may provide a broader representation of the balance between innate inflammatory activation and adaptive immune competence than NLR alone. Nevertheless, although this theoretical rationale is biologically plausible, our study did not directly compare NMLR with other leukocyte-derived inflammatory indices such as NLR, MLR, SII, or SIRI. Therefore, whether NMLR provides incremental prognostic value beyond these established biomarkers remains uncertain and should be evaluated in future comparative studies [9,10,11,15,19,20,21,23].

The approximately twofold-higher adjusted risk of all-cause mortality observed in patients in the highest NMLR quartile is broadly consistent with previous studies evaluating leukocyte-derived inflammatory indices in COPD. Li et al. also reported that elevated NMLR was independently associated with increased five-year all-cause mortality, although direct comparison of effect estimates is limited by differences in study populations, follow-up duration, biomarker categorization, and covariate adjustment. Similarly, previous studies evaluating NLR have consistently demonstrated associations between higher inflammatory cell ratios and poorer prognosis in COPD. Together, these findings support the potential prognostic relevance of leukocyte-derived inflammatory biomarkers across different clinical settings [17].

Compared with previous studies, the present work contributes several relevant elements. First, analyses were performed in a population-based primary care cohort rather than in selected hospital populations. Second, inclusion required COPD confirmed by spirometry, reducing potential diagnostic misclassification. Third, follow-up extended over ten years, and mortality was evaluated using time-to-event methods. Together, these characteristics improve the external validity and clinical relevance of the findings. Accordingly, the present findings confirm the consistency of previous observations while extending the available evidence to a large, population-based primary care cohort with spirometry-confirmed COPD and prolonged follow-up.

From a clinical perspective, the observed survival gradient across NMLR categories suggests that this biomarker may be associated with additional prognostic information beyond routinely collected clinical variables [6,7,14]. Nevertheless, the present findings should not be interpreted as evidence of predictive superiority or immediate implementation in clinical decision-making. The study did not evaluate discrimination performance, calibration, risk reclassification, or clinical utility, and therefore conclusions regarding incremental prognostic value cannot be established [17].

Several strengths should be highlighted. The study included a large real-world cohort, required COPD confirmed by spirometry, incorporated routinely available laboratory measurements, and used multivariable survival modelling with prolonged mortality follow-up. These characteristics improve reproducibility and support future external validation.

Several limitations should also be acknowledged. First, selection bias should be considered when interpreting our findings. Although the initial source population comprised 16,898 patients with a diagnosis of COPD, only those with spirometry-confirmed airflow obstruction and an available baseline complete blood count were eligible for inclusion. Consequently, the final cohort may represent patients receiving closer clinical follow-up and with a higher burden of disease or comorbidity than the overall COPD population managed in primary care. Therefore, caution is warranted when extrapolating these results to all individuals with COPD. Nevertheless, the use of spirometry-confirmed COPD strengthens the internal validity of the study by minimizing disease misclassification.

Second, the retrospective observational design precludes causal inference, and residual confounding cannot be excluded despite multivariable adjustment. Although the analyses were adjusted for several important demographic and clinical variables, other established prognostic factors in COPD (including exacerbation history, inhaled pharmacological treatment, long-term oxygen therapy, vaccination status, exercise capacity, dyspnea severity, and GOLD classification) were not consistently available in the electronic health record database and therefore could not be included in the analyses. Consequently, residual confounding cannot be completely excluded. Third, the available electronic health record dataset included mortality status and date of death but did not provide a complete and validated underlying cause of death. Consequently, we were unable to distinguish respiratory, cardiovascular, oncological, and other causes of mortality or to perform competing-risk analyses. Classification based solely on diagnoses recorded in primary care records around the time of death could have introduced substantial outcome misclassification. Future studies linking primary care data with official cause-of-death registries should evaluate whether the association between baseline NMLR and mortality differs according to specific causes of death, particularly cardiovascular and respiratory mortality. Fourth, although baseline NMLR was calculated using the most recent complete blood count available around cohort entry obtained under clinically stable conditions, blood sampling was not fully standardized. Blood counts were selected to reflect the patient’s baseline clinical status and were not intended to represent measurements obtained during hospitalization, acute infection, COPD exacerbation, or decompensation of another chronic condition. However, given the retrospective design and the use of routinely collected electronic health records, unrecognized acute inflammatory conditions, recent COPD exacerbations, or systemic corticosteroid treatment cannot be completely excluded and may have influenced leukocyte counts, NMLR values, and subsequent mortality risk. Fifth, body mass index (BMI) and Charlson Comorbidity Index were not incorporated into the primary adjusted model because of substantial missingness. However, an additional sensitivity analysis using multiple imputation yielded results consistent with those of the primary complete-case analysis, supporting the robustness of the observed association between baseline NMLR and long-term all-cause mortality. Sixth, although the continuous analysis assumed a log-linear association between log_2_-transformed NMLR and mortality, we did not formally evaluate potential non-linear relationships using flexible modelling approaches such as restricted cubic splines. Nevertheless, the consistent increase in mortality risk observed across increasing NMLR quartiles supports the robustness of the overall association. Future studies should investigate potential non-linear associations using more flexible modelling strategies. Finally, external validation in independent cohorts, together with assessment of incremental prognostic performance, remains necessary before clinical implementation can be considered [17]. Furthermore, future studies using linked and validated mortality registries should determine whether baseline NMLR is differentially associated with respiratory, cardiovascular, oncological, or other causes of death.

## 5. Conclusions

Baseline neutrophil-to-monocyte plus lymphocyte ratio (NMLR) was independently associated with long-term all-cause mortality in patients with COPD confirmed by spirometry followed in primary care. These findings support the concept that leukocyte-derived inflammatory markers may contribute to capturing prognostic heterogeneity beyond airflow limitation alone and reinforce the relevance of systemic inflammation in COPD outcomes. Because NMLR is derived from routinely available complete blood counts, it may represent a promising prognostic biomarker and warrants further evaluation for incorporation into risk-stratification models for patients with COPD. However, external validation, assessment of incremental prognostic value, and prospective studies are required before routine clinical implementation can be recommended.

## Figures and Tables

**Table 1:** Baseline characteristics of the analytic cohort according to baseline NMLR quartiles.

Characteristic	Overall	Q1	Q2	Q3	Q4	*p*-Value
Age, years	70.1 (11.5)	67.9 (11.6)	69.0 (11.6)	70.7 (11.1)	72.9 (11.2)	<0.001
Female sex, n (%)	667 (25.2)	241 (36.5)	181 (27.4)	138 (20.9)	107 (16.2)	<0.001
Current smoker, n (%)	588 (22.2)	151 (22.8)	142 (21.5)	148 (22.4)	147 (22.2)	0.947
Former smoker, n (%)	641 (24.2)	154 (23.3)	170 (25.7)	152 (23.0)	165 (25.0)	0.604
FEV1, % predicted	60.5 (21.4)	64.6 (22.0)	61.5 (20.0)	60.0 (22.1)	55.8 (20.3)	<0.001
BMI, kg/m^2^	28.4 (4.9)	28.4 (4.9)	28.4 (4.7)	29.0 (5.1)	27.7 (4.9)	<0.001
NMLR	2.20 (1.63–3.09)	1.33 (1.12–1.50)	1.89 (1.76–2.04)	2.55 (2.35–2.80)	4.47 (3.57–7.77)	—
Hypertension, n (%)	1502 (56.8)	333 (50.4)	367 (55.5)	399 (60.4)	403 (61.0)	<0.001
Diabetes mellitus, n (%)	622 (23.5)	125 (18.9)	144 (21.8)	165 (25.0)	188 (28.4)	<0.001
Ischemic heart disease, n (%)	135 (5.1)	23 (3.5)	36 (5.4)	34 (5.1)	42 (6.4)	0.117
Heart failure, n (%)	178 (6.7)	19 (2.9)	30 (4.5)	47 (7.1)	82 (12.4)	<0.001
Atrial fibrillation, n (%)	250 (9.5)	42 (6.4)	47 (7.1)	64 (9.7)	97 (14.7)	<0.001
Chronic kidney disease, n (%)	225 (8.5)	35 (5.3)	44 (6.7)	68 (10.3)	78 (11.8)	<0.001
Cancer, n (%)	372 (14.1)	78 (11.8)	73 (11.0)	101 (15.3)	120 (18.2)	<0.001
Bronchiectasis, n (%)	234 (8.9)	49 (7.4)	63 (9.5)	51 (7.7)	71 (10.7)	0.109

Values are presented as mean (SD), median (IQR), or n (%). Continuous variables were compared using one-way analysis of variance (ANOVA) or Kruskal–Wallis tests, as appropriate. Categorical variables were compared using χ^2^ tests. BMI was available at or before baseline NMLR assessment for 1009 participants (38.2%); BMI data were missing for 1635 participants (61.8%). Abbreviations: BMI, body mass index; FEV1, forced expiratory volume in one second; IQR, interquartile range; NMLR, neutrophil-to-monocyte plus lymphocyte ratio; SD, standard deviation.

**Table 2:** Mortality and survival estimates according to baseline NMLR quartiles.

Quartile	N	Deaths	Person-Years	Median Follow-Up (Years)	Mortality (%)	Rate per 100 Person-Years	5-Year Survival (%)	10-Year Survival (%)
Q1	661	187	5412.94	9.17	28.29	3.45	90.13	69.26
Q2	661	225	5142.61	9.07	34.04	4.38	84.71	63.20
Q3	661	275	4806.03	8.70	41.60	5.72	76.61	54.16
Q4	661	398	3869.75	6.33	60.21	10.28	60.37	35.92

**Table 3:** Cox proportional hazards regression models for all-cause mortality.

Model	Comparison	HR (95% CI)	*p*-Value
Model 1 (unadjusted)	Q2 vs. Q1	1.28 (1.05–1.55)	0.014
	Q3 vs. Q1	1.68 (1.40–2.02)	<0.001
	Q4 vs. Q1	3.10 (2.60–3.69)	<0.001
Model 2 (age- and sex-adjusted)	Q2 vs. Q1	1.16 (0.96–1.41)	0.127
	Q3 vs. Q1	1.42 (1.17–1.71)	<0.001
	Q4 vs. Q1	2.33 (1.95–2.78)	<0.001
Model 3 (fully adjusted)	Q2 vs. Q1	1.16 (0.95–1.41)	0.142
	Q3 vs. Q1	1.32 (1.09–1.59)	0.005
	Q4 vs. Q1	2.00 (1.66–2.40)	<0.001
Continuous NMLR (log_2_-transformed)	Per doubling of NMLR	1.34 (1.26–1.42)	<0.001

## Data Availability

The data presented in this study are not publicly available because they were derived from pseudonymized electronic health record data and are subject to ethical, legal, and institutional restrictions. Access to the datasets may be considered upon reasonable request to the corresponding author and subject to approval by the relevant data governance authorities and Research Ethics Committee, in accordance with applicable data protection regulations.

## Supplementary Materials

The following supporting information can be downloaded at https://www.mdpi.com/article/10.3390/healthcare14152431/s1, Table S1: Sensitivity analysis of the association between baseline NMLR and all-cause mortality after additional adjustment for body mass index and Charlson Comorbidity Index.
