# Supplementary material for: Peripheral Blood Neutrophil–Monocyte-to-Lymphocyte Ratio and Long-Term All-Cause Mortality in Patients with Chronic Obstructive Pulmonary Disease: A Population-Based Primary Care Cohort Study
Source: Healthcare (Basel). 2026 Aug 6;14(15):2431. doi: 10.3390/healthcare14152431 (PMC13465210; doi:10.3390/healthcare14152431)
Supplement: Supplementary file 1 [file healthcare-14-02431-s001.zip › healthcare-4427315-supplementary.pdf]

Supplementary Table S1.

Supplementary Table S1

Supplementary Table S1. Sensitivity analysis of the association between baseline NMLR and all-cause mortality after additional adjustment for body mass index and Charlson Comorbidity Index.

Variable	*Primary analysis (complete-case) HR (95% CI)*	*Sensitivity analysis adjusted for BMI and CCI HR (95% CI)*	*p value*
NMLR Quartile 2 vs Quartile 1	*1.16 (0.95–1.41)*	***1.17 (0.96–1.43)***	***0.110***
NMLR Quartile 3 vs Quartile 1	*1.32 (1.09–1.59)*	***1.34 (1.11–1.62)***	***0.003***
NMLR Quartile 4 vs Quartile 1	***2.00 (1.66–2.40)***	***2.00 (1.67–2.40)***	***<0.001***
Log_2 NMLR (continuous)	*1.34 (1.26–1.42)*	***1.33 (1.26–1.41)***	***<0.001***

Abbreviations: *BMI, body mass index; CCI, Charlson Comorbidity Index; CI, confidence interval; HR, hazard ratio; MICE, multiple imputation by chained equations; NMLR, neutrophil–monocyte-to-lymphocyte ratio.*

The primary model was adjusted for age, sex, smoking status, FEV_1 percentage predicted, hypertension, diabetes mellitus, ischemic heart disease, heart failure, atrial fibrillation, chronic kidney disease, cancer, and bronchiectasis. In the sensitivity analysis, missing BMI values were handled using multiple imputation by chained equations with 20 imputed datasets. The Charlson Comorbidity Index was reconstructed from diagnoses recorded before baseline NMLR assessment. The Cox models were additionally adjusted for BMI and Charlson Comorbidity Index, and estimates were pooled according to Rubin's rules.
